# Development of a Computerised Adaptive Testing and Equalisation Approaches to Assess Sleep and Quality of Life in Chronic Pain

**DOI:** 10.1002/ejp.70108

**Published:** 2025-08-22

**Authors:** Suzana Curcino Nogueira, Ana Mércia Fernandes, Rafael Lussani, Valquiria Aparecida da Silva, Ricardo Galhardoni, Julio Barbour, Rogério Pessoto Hirata, Gabriel Taricani Kubota, Manoel Jacobsen Teixeira, Daniel Ciampi de Andrade

**Affiliations:** ^1^ Department of Neurology, LIM‐62, Pain Center University of São Paulo São Paulo Brazil; ^2^ Institute of Health Sciences, Paulista University São Paulo Brazil; ^3^ School of Medicine University of City of São Paulo (UNICID) São Paulo Brazil; ^4^ Department of Health Science and Technology Aalborg University, Sport Sciences–Performance and Technology Research Group, Faculty of Medicine Denmark Center for Neuroplasticity and Pain (CNAP) Aalborg Denmark; ^5^ Department of Health Science and Technology, Denmark Center for Neuroplasticity and Pain (CNAP), Faculty of Medicine Aalborg University Aalborg Denmark

**Keywords:** chronic pain, computerised adaptative testing, equalisation, quality of life, sleep

## Abstract

**Background:**

Computerised adaptive testing (CAT) and equalisation are statistical approaches that mitigate questionnaire response burden by selecting individually tailored items according to previous response patterns; they facilitate comparing results across distinct instruments by providing conversion functions between their scores, respectively. However, they have seldom been specifically applied to the general chronic pain population. This study aimed at developing CAT and equalisation approaches for widely used sleep quality and quality of life (QoL) assessment instruments.

**Methods:**

This prospective cross‐sectional study included adult participants with chronic pain treated at specialised tertiary‐care clinics. Pittsburgh Sleep Quality Index (PSQI) and Insomnia Severity Index (ISI) were used for investigating sleep quality construct; and the 12‐Item Short Form Health Survey, WHOQoL‐BREF and 5 dimensions 3 levels EuroQol for QoL. CATs were developed for these two constructs based on graded model item response theory. Equalisation utilised equipercentile methodology.

**Results:**

Three‐hundred participants (54.4 ± 13.8 years‐old, female = 54.7%) with different chronic pain diagnoses, 77.3% of whom were neuropathic, were enrolled. CATs were developed for both constructs, with adequate model fit. A 5000‐response simulation demonstrated average reductions of 80.6% and 76.8% in items required to be answered for evaluating sleep quality and QoL, respectively, when compared to the total original questionnaire items. Equalisation functions were described for score conversions between WHOQoL‐BREF and 12‐Item Short‐Form Health Survey, and between PSQI and ISI.

**Conclusions:**

This initial study demonstrated the feasibility of CATs for assessing sleep quality and QoL in chronic pain populations; and provided equalisation functions between instruments widely used for these purposes. Future replication and validation are necessary to establish the generalisability of these findings.

**Significance:**

The high response burden inherent to the use of multiple validated instruments to assess quality of life (QoL) and of sleep undermines their systematic application in the assessment of chronic pain, both in daily practice and research settings. To address this gap, this initial study demonstrated the feasibility of employing computerised adaptive testing for this purpose within a population with diverse chronic pain conditions; and provided equalisation functions that allow for crosstalk between widely used QoL and sleep assessment tools.

## 1 Introduction

It is estimated that chronic pain is present in 11% to 40% of the worldwide population, interfering significantly in multiple dimensions of the individual's wellbeing and functionality, as well as exerting a heavy financial burden on healthcare systems (Cohen et al. 2021). However, its management remains challenging and frequently associated with poor results (Borsook et al. 2018; Gatchel et al. 2014). This may be partially explained by the complex multidimensional nature of this condition and its consequences, involving an intricate interplay of sociocultural, biological, psychological, and cognitive aspects (Borsook et al. 2007; Cohen et al. 2021).

Therefore, to better address it both in clinical practice and research settings, several instruments have been developed, validated, and recommended, focusing on specific chronic pain aspects (Fillingim et al. 2016; Pogatzki‐Zahn et al. 2025; Turk et al. 2006). However, the implementation of these tools is hindered by several aspects. The increased time required to apply them leads to respondents' fatigue and loss in compliance, limiting the validity of their results (Busner and Targum 2007). In daily practice, this is further complicated by the already relatively short time available for clinical assessment, as their use may result in an overload and increase in costs of healthcare systems (Busner and Targum 2007; Palese et al. 2016). The implementation of multiple assessment tools may also be impractical in the context of clinical trials (Busner and Targum 2007). Indeed, over half of published trials on several chronic pain conditions have been consistently found to underreport patient‐important outcome domains recommended by IMMPACT, including sleep, physical, and emotional functioning (Alebouyeh et al. 2024; Mazzei et al. 2020; Mulla et al. 2015), the latter two of which are central aspects of health‐related quality of life (QoL) (Turk et al. 2003). Moreover, the use of distinct validated tools across different clinical trials for assessing a specific construct, such as QoL, gives way to heterogeneity in outcome measurement, which in turn limits the comparability of their results (Mulla et al. 2015; Sachau et al. 2023).

In this context, strategies to shorten assessment and to allow for the comparison between overlapping tools are highly desirable (Turk et al. 2003). Computerised Adaptive Testing (CAT) is an innovative methodology that allows for shorter questionnaire completion times, without compromising its evaluation capability (Haley et al. 2006; Petersen et al. 2013). It is based on Item Response Theory (IRT) analytical modelling, which characterises the relationship between a questionnaire item and the underlying construct being measured (e.g., QoL, depression), assigning it a set of properties that describes its measurement performance (Reeve 2003). The system results in an interactive selection of tailored items from a questionnaire according to the individual's previous response pattern and halts the assessment when a threshold is reached, indicating that enough information has been gathered to enable the classification of the respondent as having or not a specific construct. Notably, this strategy has been successfully incorporated by the Patient‐Reported Outcome Measure Information System (PROMIS), a National Institutes of Health initiative that comprises a large set of web‐based robust patient‐reported outcome measure tools (Liu et al. 2010). Since its creation in 2004, the use of CAT‐delivered PROMIS instruments has grown significantly across studies addressing a large variety of health conditions (Teuwen et al. 2023; Tran et al. 2021; Yount et al. 2019). Meanwhile, their use has been very limited so far in the field of chronic pain, in part because they are not freely available and are costly. Furthermore, the comparison and interchangeability of results stemming from different assessment tools can be achieved through a process called equalisation (Ten Klooster et al. 2013; Kolen and Brennan 2016). This process enables the scores obtained from different questionnaires to be converted into a common metric system through a function, therefore favouring efforts to synthesise the literature, such as meta‐analyses and systematic reviews (Kolen and Brennan 2016).

Here, we describe the development of a CAT and equalisation functions for widely used sleep (i.e., Pittsburgh sleep quality index and insomnia severity index) and quality of life (QoL; i.e., 12‐Item Short‐Form Health Survey, World Health Organisation Quality of Life Assessment—Brief, and 5‐dimensions 3‐levels EuroQol) instruments, which are aimed at evaluating core domains recommended by IMMPACT in the assessment of people with chronic pain (Turk et al. 2003, 2006).

## 2 Materials and Methods

### 2.1 Study Design

This was a prospective cross‐sectional study aimed at: (i) developing a CAT, using item response theory (IRT) methodology, for widely used tools evaluating two core outcome domains of people with chronic pain: sleep quality and QoL; and (ii) providing equalisation formulae that allow for the comparison between the results of the studied tools within each of these domains. These outcomes were selected considering their importance and frequent underreporting across literature in the field of chronic pain (Alebouyeh et al. 2024; Mazzei et al. 2020; Mulla et al. 2015). QoL was defined according to the World Health Organisation criteria as the individuals' perception of their position in life in the context of the culture, value systems in which they live, and in relation to their goals, expectations, standards, and concerns (Kim 2014). The selected instruments for QoL evaluation were: 12‐item Short Form Health Survey (SF‐12), World Health Organisation Quality of Life Assessment—Brief (WHOQOL‐BREF) and 5‐dimensions 3‐levels EuroQoL (EQ‐5D‐3L). However, no well‐established definition for sleep quality exists and, therefore, this construct was defined according to Buysse's proposal: subjective satisfaction with sleep, appropriate timing, adequate duration, high efficiency, and sustained alertness during waking hours (Buysse 2014). For this construct, the following tools were used: the Pittsburgh Sleep Quality Index (PSQI) and the Insomnia Severity Index (ISI). These tools have been selected as they have been widely used in clinical research across different medical conditions (Fabbri et al. 2021; Pequeno et al. 2020).

This study was approved by the local Institution's Ethics Review Board (#2.072.694), was registered at ClinicalTrials.gov (NCT03410589), and conforms to the STrengthening the Reporting of Observational Studies in Epidemiology (STROBE) guidelines (von Elm et al. 2008), whenever applicable. All participants provided informed consent before they were enrolled in the study.

### 2.2 Participants

Participants were recruited between January and September 2019 among the patients being treated at specialised tertiary care chronic pain outpatient clinics of the Hospital das Clínicas of the University of São Paulo, Brazil. Inclusion criteria were: (i) age over 18 years, (ii) chronic pain based on the current International Association for the Study of Pain (IASP) criterion (Raja et al. 2020; Treede et al. 2019); iii. being a native Portuguese speaker. Exclusion criteria were: (i) previous medical history of cancer pain; (ii) hearing, visual, and/or cognitive impairment that prevented them from answering the study questions.

### 2.3 Sample Size

Although there is no exact measure of sample size in the literature required for calibrating CAT items, a minimum of 200 to 300 subjects is estimated to be sufficient, as long as the fit indexes to the model are maintained (Chuah et al. 2006; Şahin and Anıl 2017). Therefore, this study aimed at enrolling a minimum of 300 volunteers to obtain adequate indexes, selected through convenience sampling. Recruitment continued until this target number of participants with completed questionnaire assessments was reached.

### 2.4 Assessments

Participants were evaluated on a single visit. General demographic and clinical data regarding their chronic pain and previous medical history were obtained through a structured interview and medical records review. Sequentially, they were requested to fill in the validated scales and questionnaires described below. Participants received a brief explanation of these instruments before responding to them, and any doubts regarding them were clarified by the researchers.

#### 2.4.1 Sleep Assessment Tools

- Pittsburgh Sleep Quality Index (PSQI): This 27‐item questionnaire measures self‐reported sleep quality and disturbance over a 1‐month period, across seven components: subjective sleep quality, sleep duration, sleep efficiency, sleep disorders, use of sleep‐inducing medications, and nocturnal dysfunction. Its score ranges from 0 to 21, and higher scores indicate lower sleep quality. A score > 5 is a sensitive and specific measure of poor sleep quality (Buysse et al. 1989).
- Insomnia Severity Index (ISI): This is a seven‐item, self‐administered questionnaire that evaluates the nature, severity, and impact of insomnia over the past 2 weeks. The total score is interpreted as follows: absence of insomnia (0–7), sub‐threshold insomnia (8–14), moderate insomnia (15–21), and severe insomnia (22–28) (Bastien et al. 2001).

#### 2.4.2 Quality of Life Assessment Tools

- 12‐item Short Form Health Survey (SF‐12): This 12‐item questionnaire measures functional health and well‐being from the respondent's perspective. It provides two summary scores: the mental component (MCS) and the physical component summaries (PCS). Higher scores represent a better QoL (Ware et al. 1996).
- WHOQOL‐BREF: This 26‐item questionnaire investigates QoL across four domains: physical, psychological, social relationships, and environment. Its final score ranges from 0 to 100, with higher scores indicating better QoL (Harper et al. 1998).
- EuroQol 5‐dimensions 3‐levels (EQ‐5D‐3L): This tool is composed of two parts, of which only the first was included in this study. This part is a five‐item questionnaire that assesses QoL across the following dimensions or domains: mobility, self‐care, usual activities, pain/discomfort, and anxiety/depression. Each dimension is classified into three levels of severity: no problems (level 1), some problems (level 2), extreme problems (level 3). With this, 243 theoretically possible health states are defined, varying from 11 111 (no problems in any dimension) to 33 333 (severe problems in all dimensions) (Hurst et al. 1997).

In order to better characterise the clinical characteristics and interference associated with their chronic pain conditions, participants were also evaluated with the following pain assessment tools: Douleur Neuropathique 4 questions (DN‐4) (Bouhassira et al. 2005), Visual Analogue Scale (VAS) for average pain intensity, Brief Pain Inventory (BPI) (Cleeland and Ryan 1994), Short‐form McGill Pain Questionnaire (SF‐MPQ) (Melzack 1987), the Patient Global Impression of Improvement (PGI‐I) and the Clinical Global Impression of Improvement (CGI‐I) (Guy 1976). As this was a cross‐sectional study, the latter instrument was scored according to data available from the participant's electronic medical record. Neuropathic pain was defined as a score ≥ 4 at the DN‐4. Further details about the instruments included in this research can be found in the Supporting Information, 2–8.

### 2.5 Statistics

For descriptive analysis and sample characterisation, continuous quantitative variables were presented as absolute numbers, mean, and standard deviation; and for categorical variables, absolute frequency and percentage were used. Comparison between different sample groups was performed when appropriate, as indicated: numerical variables were compared through *t*‐tests and one‐way analyses of variance (ANOVA), and categorical variables through chi‐square tests. Comparisons for the exploratory analysis were conducted through standardised differences.

Numeric variables were categorised as follows: (a) numeric rating scale score for average pain intensity (item 5 of the BPI) was categorised into no pain (zero), mild pain (between one and three), moderate pain (between four and six), and severe pain (seven or above); (b) the PSQI questions two, three and four were split into five percentiles. We used correlation matrices and plots as exploratory analysis tools to better understand the correlation across all items. Items with a direction that was opposite to the overall construct (QoL or sleep quality) were inverted. Pearson, Polychoric, and Polyserial correlation tests were applied as appropriate. Missing data were addressed through multiple imputation by chained equations (MICE), which allows for the use of specific algorithms for each type of variable, such as predictive for continuous variables and logistic regression for binary ones (van Buuren 2018). We also conducted a series of exploratory factor analyses using oblique and orthogonal rotations to explore different factorial solutions underlying the data, using maximum likelihood as the extraction method. Our heuristic for the selection of factor solutions included scree plots solutions that were theoretically justifiable, and solutions where items were loaded with values above 0.30 on a single factor, while all other loadings were below that level. All analyses were performed using the R language (R Core Team 2021), including the R package mice for multiple imputation (van Buuren and Groothuis‐Oudshoorn 2011).

### 2.6 Item Response Theory Modelling

For the development of the CATs, the mathematical model of IRT was used. This model estimates the parameters of the items (i.e., a process called calibration), measures their relationship with the construct of interest (theta level) and generates an algorithm that allows identifying for each patient which items are important, which are redundant, and which are expendable, based on a set of responses obtained in a given sample (Kolen and Brennan 2016; Reeve 2003). In order to identify the theta level (i.e., which measures the relationship with the evaluated construct) associated with each questionnaire item, graded models were used (Samejima 1968) since all constructs presented mixed item types (dichotomous and ordinal) (Supporting Information, 9).

In this study, to facilitate the interpretation of the results, the response categories of some questions were inverted. Consequently, for the QoL and sleep quality constructs, high theta levels meant better QoL and better sleep quality. IRT modelling was evaluated using item characteristic curves (ICC). The fit of our IRT models was evaluated using the root mean square error of approximation, with the criterion for acceptable fit being set at a value < 0.10 (Fieo et al. 2016).

### 2.7 Item Selection and Differential Item Functioning Evaluation

Items were selected to integrate the sleep quality and QoL CATs, taking into consideration the presence of one‐dimensionality, local independence, and the absence of differential item functioning. The former is an IRT premise that allows for the evaluation of each item independently. The latter indicates that the response to an item could be affected by the respondent's gender or other unrelated variables, which were excluded (Supporting Information, 10–11). The detection of differential item functioning (DIF) was performed by generating parameters using the graded response model (Samejima 1968). DIF was considered to be present if there were significant group differences in the ICC resulting from these models, which reflect unequal probabilities of response, using gender as the stratification variable. The Wald test was used as the primary method to detect DIF while assessing group differences in IRT parameters (Supporting Information, 10–11).

### 2.8 Computerised Adaptive Testing

To initialize our CAT systems, the first item for each of the CATs (QoL and sleep quality) was selected at random. To select the sequence of items to be presented to patients in relation to the pain level, we used a set of selection criteria (Chalmers 2016) including: the determinant‐rule (D‐rule), the trace of the information or the asymptotic covariance matrix (T‐rule and A‐rule, respectively), the weighted composite rule (W‐rule), the eigenvalue‐rule (E‐rule), and the Kullback–Leibler divergence criteria (Mulder and Van Der Linden 2009). The IRT scoring method used maximum‐likelihood estimation, evaluation of the expected or maximum values of the posterior distribution, and weighted likelihood estimation. For the IRT analysis, missing values were handled using imputation algorithms, followed by sensitivity analyses to assess whether our results were stable irrespective of imputation.

Finally, to determine when the CAT session should be stopped, and therefore the obtained score be considered final (T‐score), multiple criteria were used, terminating the test based on the standard error of measurement, when inferences about the precision of the pain level were required. We considered a standard error of 0.3 as a cutoff to stop the CAT. No time limit nor maximum number of questions were set. The final T‐score was calculated with the formula: T−score=θx10+50.

The CAT Application Programming Interface (API) was hosted at the local REDCap server (Harris et al. 2019). The interface delivers CAT items to REDCap and computes the final CAT score for a given patient. To enable the connection between this API and REDCap, the latter was adapted through reverse engineering (Supporting Information, 12–13). This allowed for the sequence of questionnaire items being presented to the respondent to be determined according to their previous questionnaire answers. Parameter determination was carried out using the Multidimensional Item Response Theory (MIRT) package, and the CAT creation using the Computerised Adaptive Testing Multidimensional Item Response Theory (mirtCAT) package.

### 2.9 Clinical Interpretation of Theta Levels

After analysing the data, the items were organised for clinical characterisation of patients according to theta. Response categories of polytomous items were dichotomised to allow the calculation of probabilities of endorsing the response categories of each item independently for each theta level. Thus, response categories were transformed into separate items that were evaluated in isolation to observe the chance that a given patient, according to their theta, would endorse that response. To provide the clinical interpretation, the 10 most endorsed response categories were identified for each theta level, for which discrimination was at least moderate (≥ 0.65). After selecting the most endorsed items for each theta level, item synthesis was carried out to allow clinical translation of the theta obtained through the CAT. Therefore, the clinical interpretation of these levels is given by the items that compose it.

### 2.10 Equalisation of Assessment Instruments

The equipercentile score equating method (Kolen and Brennan 2016) was used to determine the relationship between the assessment instruments being equalised, and to provide a score conversion function between them. In the case of discrete scales, the cumulative distribution was estimated using percentile ranks.

## 3 Results

### 3.1 Participants and Overall Results of the Assessment Instruments

**FIGURE 1:**
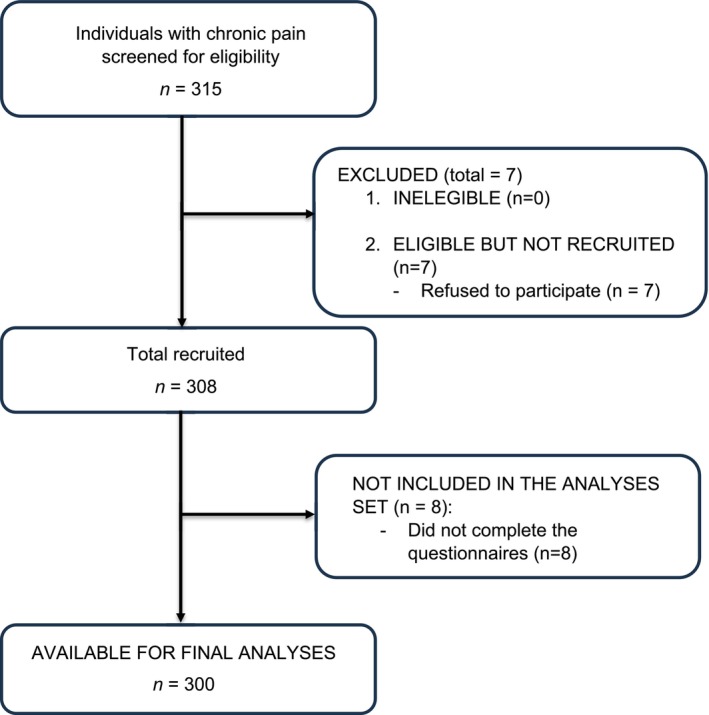
Data collection flowchart.

A total of 308 individuals were enrolled in this study, but eight participants were excluded from analyses for choosing to return incomplete data collection instruments (Figure 1). Therefore, the final analysis set was composed of 300 subjects (54.7% female), with a mean age of 54.4 ± 13.8 years old. The average pain intensity measured with VAS was 51.6 ± 31.0 mm; its mean duration was 10.9 ± 8.0 years, and its causes were: spinal cord injury (*n* = 75, 23.3%), musculoskeletal pain (*n* = 62, 20.6%), central post‐stroke pain (*n* = 37, 12.3%), and other causes (*n* = 46, 16.0%). Notably, 77.3% (*n* = 232) of participants suffered from chronic neuropathic pain. The results of the assessment tools addressing pain characteristics and interference, patient and clinician global impression of change with treatment, sleep quality, and QoL are presented in Table 1. Further details about general demographic characteristics of the sample can be found in Supporting Information, 14–15.

**TABLE 1:** Pain, sleep, quality of life assessment and, global asssessment.

	Total (*N* = 300)	Missing^A^
Pain assessment		
Douleur neuropathique 4^B^	5.6 ± 2.6 (0–10)	0
VAS for average pain intensity	51.6 ± 31.0 (0–100)	20
Brief pain inventory		0
Pain severity items (NRS)		
Worst pain in last 24 h	4.5 ± 2.5 (0–10)	0
Least pain in last 24 h	6.1 ± 2.2 (0–10)	0
Pain on average	5.7 ± 2.9 (0–10)	0
Pain right now	7.1 ± 2.3 (0–10)	0
Pain interference items (NRS)		
General activity	6.1 ± 3.1 (0–10)	0
Mood	5.0 ± 3.5 (0–10)	0
Walking ability	5.9 ± 3.5 (0–10)	0
Normal work (including housework)	6.3 ± 3.4 (0–10)	0
Relations with other people	4.3 ± 3.6 (0–10)	0
Sleep	5.8 ± 3.5 (0–10)	0
Enjoyment of life	4.6 ± 3.9 (0–10)	0
Percentage of pain relief provided by pain treatment (0%–100%)	49.3 ± 29.0 (0–100)	0
Short‐form McGill pain questionnaire		
Total score (0–15)	8.8 ± 3.9 (0–15)	0
Sensory (0–8)	4.9 ± 2.4 (0–8)	0
Affective (0–5)	2.6 ± 1.5 (0–5)	0
Evaluative (0–2)	1.2 ± 0.6 (0–2)	0
Sleep assessment		
Pittsburgh sleep quality index^C^	9.5 ± 4.9 (0–21)	0
Good sleep quality	44 (15%)	
Poor sleep quality	256 (85%)	
Insomnia severity index		0
No clinically significant insomnia (0–7)	113 (37.6%)	
Subthreshold insomnia (8–14)	84 (28%)	
Clinical insomnia (moderate severity) (15–21)	63 (21%)	
Clinical insomnia (severe) (22–28)	40 (13.3%)	
Quality of life assessment		
12‐item short form survey	82.4 ± 16.70 (50.2–177.3)	0
Mental component summary	48.5 ± 10.4 (23.8–72.1)	
Physical component summary	33.8 ± 11.0 (10.3–60.7)	
WHOQOL‐BREF	12.3 ± 2.4 (4.6–18.4)	0
Physical health	10.3 ± 3.2 (4–19.4)	
Psychological	13.1 ± 3.3 (4–19.3)	
Social relationships	13.3 ± 3.4 (4–20)	
Environment	12.4 ± 2.5 (4–19.5)	
EQ‐5D‐3L		
Mobility		33
1 = no problems	71 (26.5)	
2 = some problems	179 (67.0)	
3 = severe problems	17 (6.3)	
Self‐care		30
1 = no problems	164 (60.7)	
2 = some problems	96 (35.5)	
3 = severe problems	10 (3.7)	
Usual activities		30
1 = no problems	71 (26.3)	
2 = some problems	170 (63.2)	
3 = severe problems	28 (10.4)	
Pain/discomfort		31
1 = no problems	10 (3.7)	
2 = some problems	152 (56.3)	
3 = severe problems	108 (40)	
Anxiety/depression		30
1 = no problems	85 (31.4)	
2 = some problems	135 (50)	
3 = severe problems	50 (18.5)	
Global impression of improvement assessment		
Clinical global impression of improvement		59
Very much improved	22 (9.1)	
Much improved	43 (17.9)	
Minimally improved	38 (15.8)	
No change	95 (39.4)	
Minimally worse	20 (8.3%)	
Much worse	19 (7.9)	
Very much worse	4 (1.7)	
Patient global impression of improvement		22
Very much improved	56 (20.1)	
Much improved	55 (19.8)	
Minimally improved	66 (23.8)	
No change	64 (23)	
Minimally worse	12 (4.32)	
Much worse	21 (7.5)	
Very much worse	4 (1.4%)	

*Note:* Data presented as *N* (%) or mean ± standard deviation (min‐max).

Abbreviations: EQ‐5D‐3L, EuroQoL 5‐dimensions 3‐levels; NRS, 11‐point numerical rating scale; WHOQOL‐BREF, World Health Organisation Quality of Life Assessment–Brief.

^A^ Missing: total number of participants with missing information for each item.

^B^ Data derived from all participants included in this study, irrespective of whether they suffered from neuropathic pain or not.

^C^ Poor sleep quality was defined as a Pittsburgh Sleep Quality Index > 5.

### 3.2 Item Selection and Model Adjustment According to the Item Response Theory

For the sleep quality construct, 11 questions were excluded: 8 for violating the principle of place independence (PSQI items: 15, 16, 22, 23, 24, 25, 26, and 27), and 3 for not meeting the one‐dimensionality criteria (PSQI items: 1, 2, and 21), leaving 23 items (16 items from the Pittsburgh Sleep Quality Index and 7 from the Insomnia Severity Index) in the question bank of this construct. Meanwhile, among the QoL assessment instruments, 2 items (SF‐12 item 8 and WHOQOL‐BREF item 9) did not obtain a factor loading ≥ 0.3 and therefore were excluded, resulting in 41 items in this question bank. No items were excluded due to DIF.

After the exploratory factor analysis, data adequacy tests were performed according to the adopted graded response model with satisfactory results (Table 2).

**TABLE 2:** Fit statistics for the sleep and quality of life and constructs.

	M2	df	RMSEA (95% CI)	SRMSR	TLI	CFI
Quality of sleep	368	177	*p* < 0.001	0.069 (0.059, 0.079)	0.077	0.907
Quality of life	1.701	641	*p* < 0.001	0.1 (0.094, 0.105)	0.104	0.869

Abbreviations: CFI, comparative fit index; RMSEA, root mean square error of approximation; SRMSR, standardised root mean square error; TLI, Tucker Lewis index.

### 3.3 Analyses of Item Parameters

#### 3.3.1 Sleep Construct

Considering the two instruments for sleep assessment together, 10 (43.4%) questions presented very high discrimination, while 2 (8.6%) were classified as high, 10 (43.4%) as moderate, 1 (4.3%) as low, and none as very low. The items with the lowest discrimination coefficients were PSQI questions 5 g (“during the past month, how often you had trouble sleeping because you feel too hot?”) and 6 (“during the past month, how often have you taken medicine to help you sleep?”). The items with the highest discrimination coefficients were: ISI question 2 (difficulty staying asleep) and PSQI question 9 (“during the past month, how would you rate your sleep quality overall?”). On one hand, the item with the least difficulty was PSQI item 7 (“during the past month, how often you had trouble staying awake?”) in the answer “3 or more times a week”, which is endorsed by patients with theta as low as −2.624, indicating very poor sleep quality. On the other hand, the most difficult item was PSQI question 4 (“how many hours of actual sleep do you get at night?”) in patients who sleep more than 8 h.

#### 3.3.2 Quality of Life Construct

Regarding the ability to discriminate, 3 (7.3%) items were classified as low, 14 (34.1%) average, 9 (21.9%) high, and 15 (36.5%) very high. The items with the highest slope coefficients were: SF‐12 questions 11 (“how much of the time during the past 4 weeks have you felt downhearted and blue?”) and 10 (“how much of the time during the past 4 weeks did you have a lot of energy?”). The lowest discrimination coefficients included WHOQOL‐BREF questions 24 (“how satisfied are you with your access to health services?”) and 23 (“how satisfied are you with the conditions of your living place?”). The question with least difficulty was WHOQOL‐ BREF 23 (“how satisfied are you with the conditions of your living place?”) answer option “very dissatisfied”, while the one with greatest difficulty was WHOQOL‐ BREF 4 (“how much do you need any medical treatment to function in your daily life?”) response option “nothing”.

### 3.4 Computerised Adaptive Testing

A simulation was performed with 5000 responses, with different thetas generated by CAT using the mirtCAT R package (limited to SE < 0.3). For the sleep quality construct, the average number of questions answered in the simulation was 6.58, a reduction of 80.6% (*n* = 27.4/34) in relation to the total number of questions in the selected instruments, and of 71.3% (*n* = 16.4/23) in relation to the number of questions that make up the CAT database. When examining the scales alone, there is no decrease in the number of items for the ISI, but there was a reduction of 75.6% (*n* = 20.4/27) for the PSQI. In the QoL construct, the average number of questions answered in the simulation was 9.96, a reduction of 76.8% (*n* = 33.0/43) in relation to the total number of items in the instruments, and of 76.2% (*n* = 31.2/41) in relation to the number of questions that comprise the CAT database for this construct. Considering the individual instruments, there was a reduction of 17% for SF‐12 (*n* = 2.0/12) and of 61.6% for the WHOQOL‐ BREF (*n* = 14.8/24).

The code of the developed CATs for quality of life and sleep was made available at a public repository: https://github.com/gkubota96/CATQandS. It should be highlighted that these CATs will only indicate the set of items from each of the included questionnaires that should be answered during each assessment, as well as the order in which they should be completed. However, they will not provide the content (i.e., question and response alternatives) of each item. Access to these contents, as well as the original questionnaires themselves, should be made by the user, in accordance with the applicable legal and copyright regulations.

### 3.5 Test Precision

**FIGURE 2:**
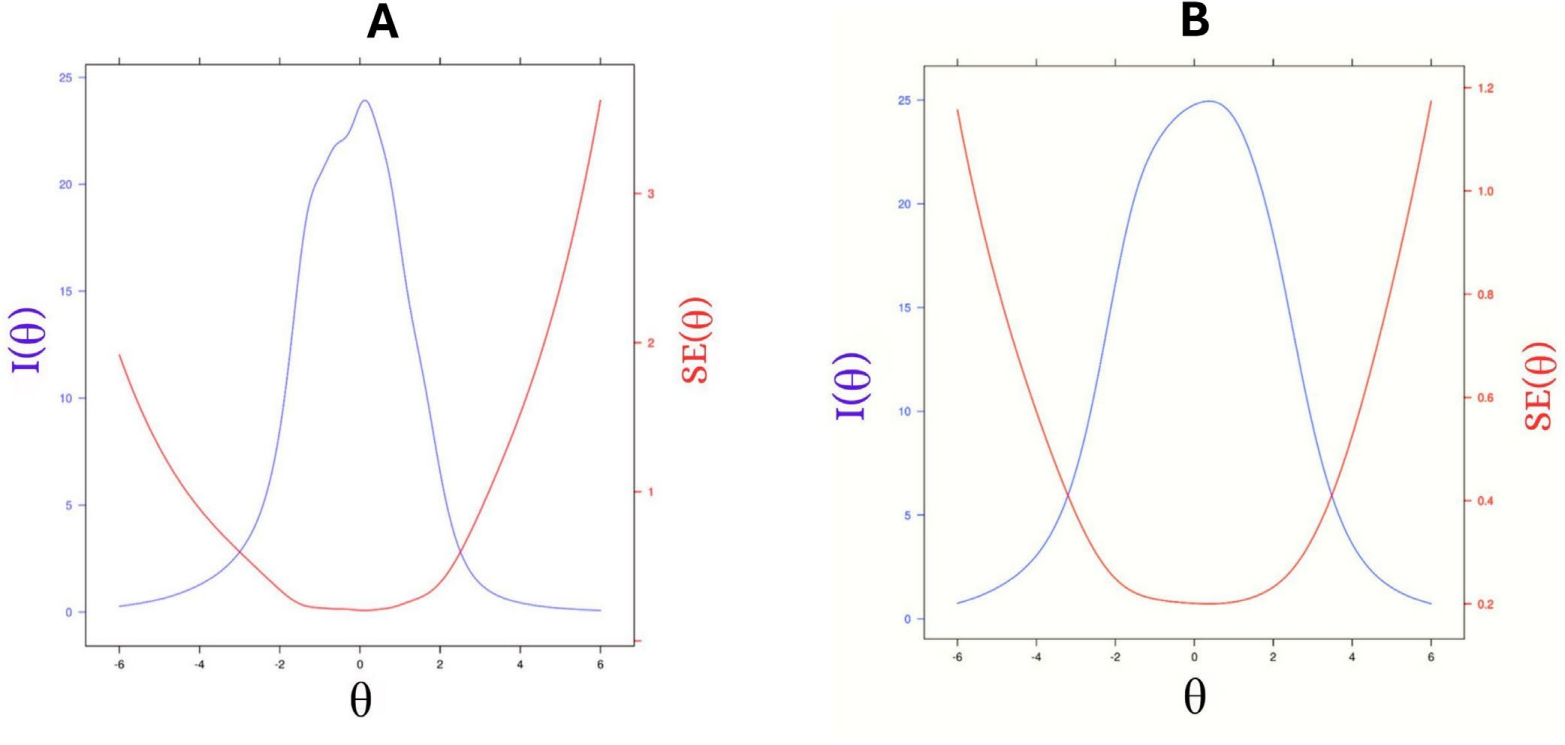
Test information and standard errors for sleep construct. These figures display the Test Information [I(θ); (blue)] and the Standard Error [SE(θ); red] curves of the Computerised Adaptive Testing (CAT) for quality of sleep (A) and quality of life (B) constructs. These curves describe the precision of the measurements provided by the developed CATs across different levels of theta (i.e., of the construct of interest). The peak of the test information curve indicates the range of theta where the test is most informative, which approaches 0 in both curves. On the other hand, the theta range between its intersection with the standard error curve indicates the theta levels across which the tests' precisions are acceptable, that is, between −3 and 2 for the quality of sleep construct (A) and between −4 and +4 for the quality of life construct (B).

The test information curves indicate that the CATs have satisfactory coverage across different theta levels of their respective construct of interest. For the sleep quality construct, this result indicated an acceptable local precision approximately between −3 and +2 theta values, with a maximum amount of information (Figure 2A); and for the QoL construct, the test information function was approximately between −3 and +3 theta values. This result indicated an acceptable local precision approximately between −4 and +4 theta values. The amount of information has a maximum at a theta level of approximately 0 (Figure 2B).

### 3.6 Clinical Interpretation of Theta Levels

The 10 most endorsed items for each theta level were synthesised to allow a clinical translation of the theta obtained through the test characteristic curve. Higher theta levels indicate better QoL or sleep quality. This clinical interpretation is presented in Table 3 for the sleep quality construct, and in Table 4 for the QoL construct.

**TABLE 3:** Classification of theta levels for the quality of sleep.

Classification	Clinical features
Very poor (θ = −2)	Assesses sleep quality as very poor, being very dissatisfied with sleep. People around perceive significantly that sleep‐related issues disrupt daily routine in an extremely important way. Presents, on most days: great difficulty initiating and maintaining sleep, difficulty sleeping due to pain, use of sleep medications, and difficulty maintaining enthusiasm in daily activities
Poor (θ = −1)	On most days, experiences significant difficulty initiating and maintaining sleep, use of sleep medications, waking up to use the bathroom, and difficulty maintaining enthusiasm in daily activities. Sleep problems disrupt daily routine significantly
Moderate (θ = 0)	Experiences moderate difficulty in maintaining sleep, with frequent nighttime awakenings. Sleep problems stem from pain‐related issues and various other reasons
Good (θ = 1)	Rates sleep quality as good, is not concerned about sleep, and does not experience difficulty in maintaining sleep
Very good (θ = 2)	Do not experience any difficulty in initiating or maintaining sleep. They are not concerned about this issue

**TABLE 4:** Classification of theta levels for the quality of life construct.

Classification	Clinical features
Very poor (θ = −2)	The subject considers his/her health to be bad, with extreme impairment to carrying out activities due to pain. The subject is very dissatisfied with his/her ability to work and carry out day‐to‐day activities. Never feeling energetic, feeling anxious, depressed and impaired in social activities by emotional problems all the time
Poor (θ = −1)	The subject considers his/her health to be bad, with impairment to carrying out day‐to‐day activities. Very dissatisfied with the ability to work. Has low energy, partial difficulties with mobility and self‐care. Moderate feelings of anxiety and depression
Moderate (θ = 0)	The subject considers health and quality of life to be regular, with pain that is limiting. The subject has no difficulty in self‐care, but has some difficulties in mobility and carrying out usual activities. Sometimes the subject experiences lack of energy and negative feelings. Moderate feeling of anxiety and depression. The subject considers that he/she needs medical treatment to live
Good (θ = 1)	The subject considers quality of life to be good and is satisfied with it. Some mobility problems, no impact on self‐care. Eventually he/she experiences negative feelings, but this does not result in impairment in carrying out activities. The subject does not have feelings of anxiety and depression. Good ability to concentrate

### 3.7 Assessment Instruments Equalisation

The functions generated for equalisation of the scores obtained from the studied sleep quality assessment tools were:

- ISI conversion to PSQI: =ROUND(0.7713 + ISI score × 0.6056;0)
- PSQI conversion to ISI: = ROUND(−1.101 + PSQI score × 1.632:0)

Additionally, equalisation functions produced for the included QoL instruments were:

- WHOQOL‐BREF conversion to SF‐12 PCS subscore: =ROUND(−8.716 + WHOQOL‐BREF score × 3.759;0)
- SF‐12 PCS subscore conversion to WHOQOL‐BREF: =ROUND(2.5753 + PCS subscore × 0.2585;0)
- WHOQoL‐ BREF conversion to SF‐12 MCS subscore: =ROUND(6.545 + WHOQOL‐BREF score × 3.608;0)
- SF‐12 MCS subscore conversion to WHOQOL‐BREF: =ROUND(−1.5458 + MCS subscore × 0.2715;0)

In order to operationalise the scale equalisation individually or using the EXCEL database, an open access application was developed and made available at: https://tinyurl.com/Curcino.

## 4 Discussion

Here we report the successful development of a CAT and equalisation functions for widely used sleep quality and QoL assessment tools within a sample of people with chronic pain conditions (Mazzei et al. 2020; Mulla et al. 2015). The basic assumptions for the use of IRT (i.e., one‐dimensionality and local independence) were satisfied, as well as the adjustment indexes to the model. Items that presented low or very low discrimination (PSQI item 5 g, and WHOQOL‐BREF items 23 and 24) could be removed from the instruments without significant harm if abbreviated scales for conventional application were developed. In practice, low discrimination means that the item is not able to effectively differentiate between individuals with high and low levels of the construct of interest and, therefore, may not be useful for clinical evaluation, nor for making accurate predictions or diagnoses (Chalmers 2016; Petersen et al. 2016). However, since CATs are electronically based, low and very low discrimination items can still be maintained in their question banks, as they will only be presented in the few instances when their use will add to the discrimination performance of the adaptive questionnaire (Haley et al. 2006).

Furthermore, the simulation of the developed CAT with 5000 responses evidenced a significant reduction in the number of questions required for the estimation of each construct, with the largest decrease for sleep quality. Another important aspect to be considered is that the quality of the assessment tends to improve, when compared to the conventional method of application of the studied instruments, because the CAT selects “higher quality” items according to the respondent's previous answers (Petersen et al. 2016; Reeve 2003). In other words, through the IRT, it was possible to identify the discrimination properties of each item and the difficulty of its response categories, and select the most suitable for each theta range, which then adds “the best of each instrument” (Reeve 2003). For example, if a given respondent has already demonstrated to have high QoL (i.e., high theta level) through their previous response pattern, the CAT will present a question that provides higher discrimination among individuals with high QoL, as this will contribute best to the assessment of this construct.

Our results support previous findings that indicate that CATs lead to a significant reduction in data collection burden without loss of the ability to identify the construct of interest. This has been previously demonstrated in a number of clinical settings, including: people with suicide risk (Bryan et al. 2017), arthritis (Kosinski et al. 2006), and lumbar spine impairments (Hart et al. 2006). Also, in the field of chronic pain, CATs have been developed for the assessment of specific populations suffering from headache (Ware et al. 2003) and cancer pain (Lai et al. 2005; Petersen et al. 2016), with similar results. However, as they were developed and validated within samples of individuals suffering from specific chronic pain conditions, their generalisability may be limited. Furthermore, most of these have only addressed specific pain‐related features and impact (Lai et al. 2005; Ware et al. 2003), and the only ones to have investigated health‐related QoL were limited by the exclusion of 44% of the sample who answered “no” to all pain intensity assessment questions (Petersen et al. 2016). In an effort to encourage the use of patient‐reported outcomes in clinical practice, the National Institutes of Health created a web‐based CAT (www.assessmentcenter.net) development tool and item banks, called the Patient Outcome Measurement Information System (PROMIS) (Yount et al. 2019). Some CATs are available online, including pain, fatigue, anxiety, sleep, and functionality (Teuwen et al. 2023; Victorson et al. 2019; Yount et al. 2019). Although this initiative represents a milestone for the incorporation of this approach in the field of healthcare, its items were calibrated using the general population and, to the best of our knowledge, have not been validated within the population with chronic pain (Liu et al. 2010). Therefore, the CATs developed in this study may be useful to fill in the gap of assessment across different clinical conditions, but still within the unique context of chronic pain.

In the health system there is a trend towards digitalization of information for users (Delice et al. 2023). Potentially the CATs can be transformed into mobile applications that can be answered periodically to increase the frequency of evaluation of constructs of interest (Triantafillou et al. 2008). In this sense, here we provided a free‐open use version of a web‐based app that can help to fill in this gap. This may provide more consistent data to clinicians, once serial evaluations tend to be more reliable than sporadic assessments. In addition to testing the immediate result, the CAT can also provide graphics or images that allow the physician to identify trends, discuss more objectively, and compare the results of treatment over time (Carlo et al. 2021; Triantafillou et al. 2008). Periodic self‐assessment with CATs can also be empowering for the patients, as it may foster education and adherence to treatments that require changes in attitudes and beliefs, such as chronic pain (Carlo et al. 2021). It may also be possible to create systems of indicators that monitor and alert the care team when one of the parameters shows change, allowing for earlier, more interactive, flexible, individualised, and effective interventions.

Barriers to the implementation of CATs for PRO assessment should be considered at this stage. These may include the unavailability of computerised devices in low‐resource settings and flawed patient privacy policies regarding the collection and storage of electronic data (Kane et al. 2021; Reeve 2003; Rose et al. 2012). Another disadvantage of CATs, from the perspective of the test participants, is that they are not allowed to skip or return to items already administered to change their answer, which is always possible with the conventional strategies for the administration of assessment tools (Kane et al. 2021; Petersen et al. 2016; Rose et al. 2012).

Besides the development of the CATs for sleep quality and QoL evaluation, this study was also able to determine equalisation functions that allow for the crosstalk of results obtained from the main investigated assessment tools. Equalisation is a method that allows for converging items from different scales to the same metric (McHorney and Cohen 2000). While this has been widely used in the field of education, its application to healthcare is still incipient. We used the equalisation by single group, which is the approach with the highest reliability to this process, as the two groups respond to all the instruments that will be equalised (Dorans 2007). Even though the patients were required to answer a greater number of questions, this did not limit the feasibility of the study. The symmetry property, defined as the possibility of exchanging the scores obtained between the different scales, was satisfied.

However, it is possible that an individual with a certain score on one instrument will have a different one from that determined by the equalisation table on the other, considering the rounding of values. Also, it must be considered that two individuals with identical scores on one tool may have different scores on the second. Equalisation would only correlate perfectly if the scales were identical, in which case the equalisation would not need to be done. For this reason, it is not recommended to use these tables with individual patients, but rather with groups of 20 individuals or more (Ten Klooster et al. 2013; Victorson et al. 2019). Still, equalisation proves to be useful for comparing results from studies that made use of distinct instruments, and especially for jointly analysing several databases. This may foster efforts to synthetise the ever‐growing body of evidence for chronic pain treatments, which ultimately lay the foundations for clinical practice guidelines.

It should be highlighted that the results from this study must be interpreted taking into consideration its limitations. Firstly, although it aimed at sampling a population suffering from diverse chronic pain conditions, it only included individuals being treated at specialised tertiary‐care outpatient clinics. This may have led to a higher‐than‐average prevalence of neuropathic pain (i.e., 77.3%), as enrollment included specialised spinal cord, post‐stroke, and cancer‐related pain units, within which contexts this type of pain may occur in more than half of individuals (Burke et al. 2017; Couceiro et al. 2018; Liampas et al. 2020). These aspects may have limited the generalisability of our findings. Nonetheless, it should be noted that other general demographic features of the study sample are similar to those of previously published large cohorts of individuals with chronic pain (Breivik et al. 2006; Johannes et al. 2010). Also, this was a cross‐sectional study and, consequently, the usefulness of the developed CATs for assessing variations in QoL and sleep quality constructs throughout the time remains to be evaluated. Another limitation is that the developed CAT only assessed sleep quality and QoL, and not other frequently underreported patient‐relevant outcome measures. Additionally, average pain intensity was measured with a VAS without a specific time frame, which may reduce the reliability of results for this secondary outcome. Finally, despite providing a more realistic equating between the studied instruments, the selected equipercentile method for determining the equalisation functions is more prone to sampling errors when larger numbers of parameters are considered.

## 5 Conclusions

This study led to the development of a CAT for widely used instruments for assessing sleep quality and QoL across diverse chronic pain conditions, as well as equalisation functions between important tools within these two constructs. These may help improve data collection processes and provide a more comprehensive assessment for future research among people with chronic pain, and allow for the exchangeability of scores obtained by distinct instruments within and across different studies. The scarcity of other similar studies such as this work makes it innovative and, at the same time, compromises the comparison with previously published results. Although the replication and validation of findings are necessary within larger multicentric cohorts of patients, with a more representative distribution of chronic pain conditions, this study represents an initial stage for the clinical application of computerised adaptive scales in the field of chronic pain research.

## Author Contributions

This study was designed by Suzana Curcino Nogueira and Daniel Ciampi de Andrade The experiments were performed by Suzana Curcino Nogueira and Rafael Lussani. The data were analysed by Julio Barbour, Ricardo Galhardoni, and the results were critically examined by all authors. Suzana Curcino Nogueira and Ana Mércia Fernandes had a primary role in preparing the manuscript, which was edited by Valquiria Aparecida da Silva, Rogério Pessoto Hirata, Gabriel Taricani Kubota, Manoel Jacobsen Teixeira, and Daniel Ciampi de Andrade All authors have approved the final version of the manuscript and agree to be accountable for all aspects of the work.

## Conflicts of Interest

The authors declare no conflicts of interest.

## Supporting information


**Data S1:** ejp70108‐sup‐0001‐DataS1.docx.

## Data Availability

The data that support the findings of this study and the analysis R scripts are available upon reasonable written request.
