# Supplementary material for: Development of a Computerised Adaptive Testing and Equalisation Approaches to Assess Sleep and Quality of Life in Chronic Pain
Source: Eur J Pain. 2025 Aug 22;29(9):e70108. doi: 10.1002/ejp.70108 (PMC12372430; doi:10.1002/ejp.70108)
Supplement: Supplementary file 1 — Data S1: ejp70108‐sup‐0001‐DataS1.docx. [file EJP-29-0-s001.docx]

**Supplementary files**

**Summary**

[1. Instruments and tools included in the study 2](#_Toc194739281)

[2. Graded item response theory 9](#_Toc194739282)

[3. Differential item functioning 10](#_Toc194739283)

[4. Application Programming Interface development 12](#_Toc194739284)

[5. Supplementary table 1 14](#_Toc194739285)

[6. References 16](#_Toc194739286)

# **Instruments and tools included in the study**

The following questionnaires and scales were included in this study:

1. **Quality of sleep assessment**

*Pittsburgh Sleep Quality Index*

This questionnaire was designed to provide a comprehensive overview of the various factors that influence sleep, allowing healthcare professionals to gain a detailed understanding of the patient's situation respondent's sleep over a one-month period. It is composed of 19 self-reported items, which are grouped into 7 components: subjective sleep quality, sleep latency, sleep duration, habitual sleep efficiency, sleep disturbances, use of sleep medications, and daytime dysfunction. Additionally, this tool includes 5 questions rated by the respondent’s roommate or bed partner (if applicable), solely for clinical information, that are not used in the scoring process (Bertolazi et al., 2011; Buysse et al., 1989). The global questionnaire score ranges from 0 to 21. The higher the score, the poorer the sleep quality. A score of 5 or higher has been established to indicate poor sleep quality (Buysse et al., 1989; Mollayeva et al., 2016).

*Insomnia Severity Index*

This is a self-administered questionnaire that is aimed at characterizing the severity of insomnia symptoms based on the insomnia criteria defined by the fourth edition of the Diagnostic and Statistical Manual of Mental Disorders (American Psychiatric Association, 1994), including: difficulty falling and staying asleep, as well as early awakening. It also evaluates the consequences of insomnia on daily activities and its impact on the quality of life (Bastien et al., 2001). It comprises seven Likert scale questions, with 5 points, and its total score ranges from 0 to 28. It allows for the classification of the respondent as having: absence of clinically significant insomnia (scores 0 to 7), mild insomnia (scores 8 to 14), moderate insomnia (scores 15 to 21), and severe insomnia (scores 22 to 28) (Bastien et al., 2001).

1. **Quality of life assessment**

12-item Short Form Health Survey

This is an abbreviated version of the 36-item Short Form Survey, which was developed to provide an overall assessment of the individual’s health-related quality of life, and has been used in various clinical and research contexts (Ware et al., 1996). It consists of 12 self-report Likert scale items, which evaluate eight health domains over the past four weeks, including: physical functioning, role-physical, bodily pain, general health, vitality, social functioning, role-emotional and mental health (Ware et al., 1996). Each domain is assessed by one or two items (Ware et al., 1996). A scoring algorithm derives two summary subscales from this instrument: the Physical Component Summary (PCS) and the Mental Component Summary (MCS). Each summary subscale is obtained from the scores of 6 items. The raw scores of these items are computed and normalized to produce the final PCS and MCS scores, each ranging from 0 to 100, with no established cutoff (Ware et al., 1996). Higher scores are associated with better quality of life (Ware et al., 1996).

WHOQOL-BREF

This is a reduced version of the WHOQOL-100, a questionnaire developed by the World Health Organization (WHO) to measure quality of life in a comprehensive manner, taking into account emotional, social, and environmental aspects that influence an individual's well-being (Fleck et al., 2000; Harper et al., 1998). It consists of 24 items covering four domains: physical, psychological, social relationships, and environment; as well as 2 items evaluating the individual’s overall perceptions of their quality of life and health, respectively. Each question is answered using a 5-point Likert scale, where the respondent can indicate the frequency or intensity of their experience (Fleck et al., 2000; Harper et al., 1998). Domain scores are calculated as the mean score of all items which comprise each of them, and are scaled positively, with higher values indicating higher quality of life. Domain scores are then multiplied by 4 and subsequently transformed to a 0-100 scale. Likewise, higher final values are associated with better levels of quality of life, with no established cutoff (Fleck et al., 2000; Harper et al., 1998).

EuroQol 5-Dimensions 3-Levels

Developed by the EuroQol Group, this is a validated instrument for measuring quality of life, divided in two parts. The first one is comprised by 5 items, each one assessing a specific domains: mobility, self-care, pain/discomfort, usual activities, and anxiety/depression (Hurst et al., 1997; Rabin and De Charro, 2001; Santos et al., 2016). These items offer three response categories: no problems (level 1), some problems (level 2), or extreme problems (level 3). The combination of responses for each of the five domains can define 243 distinct health states. The second part consists in a visual analogue scale evaluating the respondents general perception of their health today, anchored at 0 (worst that can be imagined) and 100 (best that can be imagined) (Hurst et al., 1997; Rabin and De Charro, 2001; Santos et al., 2016).

1. **Pain characteristics assessment**

Visual Analogue Scale for Average Pain Intensity

This is one of the most widely and simplest unimodal instrument to assess the intensity of pain in distinct time spams (e.g., past week, past 24 hours, now) (Downie et al., 1978). In this study, this scale was used to evaluate the pain intensity on average experienced by the participant, without a specific time frame. This has been described in previous studies measuring the typical or average pain intensity among individuals with long-standing chronic pain(Johnson, 2005; Korff et al., 1993; Underwood et al., 1999), and was selected to allow for a better comparison with pain intensity measured by the Brief Pain Inventory item 5 (Cleeland and Ryan, 1994), which also assesses pain intensity on average, without a specific time span, through a 11-point numeric rating scale. It can be applied quickly and does not require specialized equipment or training. It consists of a 10-centimeter straight line, where one end represents no pain (score 0) and the other represents maximum pain (score 10) (Downie et al., 1978; Price et al., 1983). The respondent is required to mark the point in this line that best describes their pain. Subsequently, the distance between the marked point and the beginning of the line (no pain extremity) is measured to determine the final score, which is usually expressed in millimeters (Downie et al., 1978; Price et al., 1983).

Brief Pain Inventory

This multidimensional instrument is comprised of 9 items, which provide a general characterization of the respondent’s pain, including: location, intensity at different time points (average, worst and least in the past 24 hours, and at the moment of the evaluation), pain relief with treatment, and its interference across several aspects of life: general activity, mood, walking ability, normal work, relations with other people, sleep and enjoyment of life (Cleeland and Ryan, 1994; Ferreira et al., 2011). Two summary scores can be derived from this tool: pain severity index and the pain interference index. The former is the arithmetic mean of the items evaluating pain intensity (items 3 to 6), and the second of the items assessing interference (Cleeland and Ryan, 1994; Ferreira et al., 2011).

Short Form McGill Pain Questionnaire

This instrument was developed to provide a general assessment of pain, within two dimensions: sensory and affective. It consists of 15 pain quality descriptors (4 affective and 11 sensory), which can be ranked by the respondent according to their intensity as 0 (none), 1 (mild), 2 (moderate) or 3 (severe) (Ferreira et al., 2013; Melzack, 1987). This tool provides 3 scores, which are derived from the sum of the intensity rank values for the sensory, the affective, and both (total) groups of descriptors, respectively. This tool has been a widely used scale for pain assessment and can be applied to patients with either neuropathic or non-neuropathic pain (Ferreira et al., 2013; Melzack, 1987).

Douleur neuropathique 4 questions

This scale was originally developed and validated in France to differentiate neuropathic pain from nociceptive pain (Bouhassira et al., 2005; Santos et al., 2010). It consists of 4 questions, that encompass: 7 self-report items which assess pain quality and associated symptoms, and 3 items which are evaluated through a simple physical examination (i.e., touch and pinprick anesthesia or hyposthesia, and dynamic mechanical allodynia). Each is scored as 0 (absent) or 1 (present). A total score is obtained by the sum of all 10 items, and indicates neuropathic pain when equal or greater than 4 (Bouhassira et al., 2005; Santos et al., 2010). This tool has demonstrated good sensitivity (83%) and specificity (90%) in detecting neuropathic pain (Bouhassira et al., 2005; Timmerman et al., 2017).

1. **Global Impression assessment**

Clinical Global Impression of Improvement Scale

This is a simple instrument that assess global and subjective impression of the healthcare provider (i.e., clinician) regarding the respondent’s (i.e., patient) improvement with the treatment since their last medical visit. It consists of a single Likert scale, with the following options: 1 = much better since the start of treatment; 2 = better; 3 = slightly better; 4 = neither better nor worse; 5 = slightly worse; 6 = worse; 7 = much worse (Busner and Targum, 2007). Since this is a cross-sectional study, information recorded in the participant’s medical records was used to complete this questionnaire.

Patient Global Impression of Improvement Scale

This scale is similar to the Clinician Impression of Improvement Scale, except it takes in consideration the patient’s point of view. Therefore, it assesses the global subjective impression of the respondent (i.e., patient) regarding their improvement with the treatment, since their last medical visit. It also consists of a 7-point Likert scale, in which the patient can select from the following options: 1 = much better; 2 = better; 3 = slightly better; 4 = neither better nor worse; 5 = slightly worse; 6 = worse; 7 = much worse (Ferguson and Scheman, 2009).

# **Graded item response theory**

The graded response model (Samejima, 1968) is an IRT model developed to evaluate surveys with ordinal responses such as ordered Likert-type scales. This model characterizes item functioning via two parameters for each item: discrimination and difficulty. Discrimination parameters evaluate how well an item discriminates (or differentiates) between individuals scoring high and low on a construct. Item difficulty (or item location) describes how high on a construct typically is before they endorse an item (Kirisci et al., 1996). In the item parameters analysis, it should be considered that the items may have positive discrimination parameters (when an increase in theta means an increase in the assessed construct) or negative discrimination (when a decrease in theta means a decrease in the measured latent trait).

# **Differential item functioning**

The main concept behind differential item functioning (DIF) is that an item should exclusively measure theta levels, its response not being affected by extraneous factors, such as sex or ethnicity. Examining differential item functioning (DIF) is important since if an item is not exclusively measuring latent trait level, it should be excluded from the item bank. A large proportion of items with differential item functioning (DIF) decreases our ability to measure the level of latent traits based on CAT scores (Van Nispen et al., 2011).

In this study, the differential item functioning (DIF) testing followed a standardized sequence (Van Nispen et al., 2011). First, we provided our internal data science group with instructions containing the DIF definition along with clinical examples. Then, experts generated DIF hypotheses, indicating for each item whether or not they expected it to be present, as well as its direction with respect to gender. Additional groups were added depending on the input from experts, including educational level. In these clinical examples, persons from each of the two groups where DIF was hypothesized to be present (eg, males versus females) should have the same likelihood of reporting difficulty in an item. If data scientists did not think this was the case, we hypothesized that the item in question could have DIF.

The detection of DIF was performed by generating parameters using the graded response model (Samejima, 1968). The item characteristic curve (ICC) resulting from these models describes the relationship between the pattern of the response of a given item and the underlying construct. There are two properties of the ICC for the graded response model: the item difficulty or location parameters, and the discrimination, which the steepness of the curve or the degree to which the item is related to the underlying attribute measured. We considered DIF to be present if there were significant group differences in the ICC, reflecting unequal probabilities of response, given equal levels of pain between the two groups. The Wald test was used as the primary method to detect DIF while assessing group differences in IRT parameters. In this process, the model was established in which all parameters were constrained to be equal across the two groups for a given item, while the target item parameters were freed to be estimated separately for the groups the experts were hypothesized to have DIF. Adjustments were made for multiple comparisons.

We conducted sensitivity analysis for DIF detection using an iterative ordinal logistic regression IRT framework (Crane et al., 2006). We performed an ordinal (common odds-ratio) logistic regression using IRT theta estimates as the conditioning variable. The generalized partial credit model was used for IRT trait estimation. Items evaluated for potentially having DIF were treated as unique items for each group to be calibrated separately, and group-specific item parameters were obtained. Items without DIF were served as anchors for IRT calibration. The procedure runs iteratively until the same set of items has been flagged over two consecutive iterations unless anchor items are specified in advance.

# **Application Programming Interface development**

The Application Programming Interface (API) system delivers CAT items to REDCap (Harris et al., 2019), also computing the final CAT score for a given patient. This description is split into three sections: architecture, security, and software dissemination.

The architecture is based on the successful model established by the Patient-Reported Outcomes Measurement Information System (PROMIS) (Liu et al., 2010), which is a CAT system that can connect to REDCap (https://www.project-redcap.org/). To develop the API connecting the developed CAT to REDCap, the architecture currently in use by PROMIS was examined and followed a similar model. As part of the preliminary work to evaluate the system feasibility, all PROMIS’ endpoints were tested and were found able to successfully connect the preliminary API system to REDCap. To extend the initial pilot project, endpoints were then changed in the backend database, ultimately making the connection between the CAT and the REDCap system seamless and fully functional. Of importance, since the code for REDCap is not open source, no changes were made to its underlying code, the only ones being made to the back end relational database (MySQL https://www.mysql.com/, an open source software) which is not part of the REDCap software. This architecture respects the proprietary nature of REDCap. While the scores are being computed, we temporarily stored all response information in a cache inside the same relational databases (MySQL) living inside a secure institutional firewall. Once patients completed their questionnaire, all information is deleted from the database and stored exclusively in REDCap along with the remaining study database. This restricted data storage is therefore consistent with the intent to maximize security and privacy. Last, to ensure low latency and avoid lags in the data entry process (i.e., the time between the response to an item and the system providing the next item), REDCap application and the API were placed in the same secure institutional server, thus reducing the data transmission to a matter of microseconds.

Regarding distribution, the CAT software is deployed through a Docker open source container (https://www.docker.com/), making it possible for information technology staff to reproduce the exact environment recommended for installation regardless of the underlying hardware and operating system available at each institution. The user interface is the one available in REDCap.

The CAT API simulates the PROMIS communication with RedCAP, but in a local environment in which we are able to run custom adaptive forms:

1. Change PROMIS URL in RedCap MySQL:

UPDATE redcap_config SET value = 'http://127.0.0.1:5234/' WHERE field_name = 'promis_api_base_url';

Original API: https://www.redcap-cats.org/promis_api/

Change the user permissions:

GRANT SELECT, INSERT, UPDATE, DELETE, CREATE, ALTER, DROP ON redcap.* TO redcap@'%' IDENTIFIED BY '#####';

This will cease any RedCAP communication to PROMIS.

The only change needed is the update of PROMIS URL in RedCAP database to the CAT API location, no change in the source code is required.

# **Supplementary table 1**

**Table S1. Demographical profile and baseline characteristics of subjects included in the study.**

|  | **Total**  **(N= 300)** | **Missing** |
| --- | --- | --- |
| Age (years) | 54.4 ± 13.8 (23-91) | 0 |
| Sex |  | 0 |
| *Female* | 164 (54.7) |  |
| *Male* | 136 (45.3) |  |
| Marital status |  | 20 |
| *Single*  *Married*  *Other* | 79 (26.3) |  |
|  | 161 (53.7) |  |
|  | 40 (13.3) |  |
| Employment status |  | 8 |
| *Employed*  *Retired*  *Others* | 84 (28) |  |
|  | 147 (49) |  |
|  | 61 (20.3) |  |
| Education |  | 104 |
| *Elementary*  *Middle*  *High school*  *College* | 111 (37) |  |
|  | 97 (32.3) |  |
|  | 82 (27.3) |  |
|  | 6 (2) |  |
| Ethnicity |  | 46 |
| *White* | 165 (55) |  |
| *Black* | 24 (8) |  |
| *Other* | 65 (21.7) |  |
| Pain duration (y) | 10.9 ± 7.98 (0.5-42) | 0 |
| Paint treatment duration (y) | 6.88 ± 5.68 (0-30) | 0 |
| Activities impared by pain |  |  |
| *Home activities* | 213 (71) |  |
| *Leisure activities* | 203 (67.6) |  |
| *Loss of working days* | 148 (49.3) |  |
| *Resulted in sick leave* | 140 (46.6) |  |

^A^ Values are presented in absolute numbers (%) ou mean ± SD (minimum and maximum);

y: years, missing: total number of participants with missing information for each item.

# **References**

American Psychiatric Association (1994). *Diagnostic and Statistical Manual of Mental Disorders* (American Psychiatric Association).

Bastien, C.H., Vallières, A., Morin, C.M. (2001). Validation of the insomnia severity index as an outcome measure for insomnia research. *Sleep Med* 2, 297–307.

Bertolazi, A.N., Fagondes, S.C., Hoff, L.S., Dartora, E.G., da Silva Miozzo, I.C., de Barba, M.E.F., Menna Barreto, S.S. (2011). Validation of the Brazilian Portuguese version of the Pittsburgh Sleep Quality Index. *Sleep Med* 12, 70–75.

Bouhassira, D., Attal, N., Alchaar, H., Boureau, F., Brochet, B., Bruxelle, J., Cunin, G., Fermanian, J., Ginies, P., Grun-Overdyking, A., Jafari-Schluep, H., Lantéri-Minet, M., Laurent, B., Mick, G., Serrie, A., Valade, D., Vicaut, E. (2005). Comparison of pain syndromes associated with nervous or somatic lesions and development of a new neuropathic pain diagnostic questionnaire (DN4). *Pain* 114, 29–36.

Busner, J., Targum, S.D. (2007). The clinical global impressions scale: applying a research tool in clinical practice - PubMed. *Psychiatry (Edgmont)* 4, 28–37.

Buysse, D.J., Reynolds, C.F., Monk, T.H., Berman, S.R., Kupfer, D.J. (1989). The Pittsburgh Sleep Quality Index: a new instrument for psychiatric practice and research. *Psychiatry Res* 28, 193–213.

Cleeland, C.S., Ryan, K.M. (1994). Pain assessment: global use of the Brief Pain Inventory. *Ann Acad Med Singap* 23, 129–138.

Crane, P.K., Gibbons, L.E., Jolley, L., Van Belle, G. (2006). Differential item functioning analysis with ordinal logistic regression techniques. DIFdetect and difwithpar. *Med Care* 44.

Downie, W.W., Leatham, P.A., Rhind, V.M., Wright, V., Branco, J.A., Anderson, J.A. (1978). Studies with pain rating scales. *Ann Rheum Dis* 37, 378–381.

Ferguson, L., Scheman, J. (2009). Patient global impression of change scores within the context of a chronic pain rehabilitation program. *J Pain* 10, S73.

Ferreira, K.A., Teixeira, M.J., Mendonza, T.R., Cleeland, C.S. (2011). Validation of brief pain inventory to Brazilian patients with pain. *Support Care Cancer* 19, 505–511.

Ferreira, K.A.S.L., de Andrade, D.C., Teixeira, M.J. (2013). Development and validation of a Brazilian version of the short-form McGill pain questionnaire (SF-MPQ). *Pain Manag Nurs* 14, 210–219.

Fleck, M.P.A., Louzada, S., Xavier, M., Chachamovich, E., Vieira, G., Santos, L., Pinzon, V. (2000). Aplicação da versão em português do instrumento abreviado de avaliação da qualidade de vida “WHOQOL-bref.” *Rev Saude Publica* 34, 178–183.

Harper, A., Power, M., Orley, J., Herrman, H., Schofield, H., Murphy, B., Metelko, Z., Szabo, S., Pibernik-Okanovic, M., Quemada, N., Caria, A., Rajkumar, S., Kumar, S., Saxena, S., Chandiramani, K., Amir, M., Bar-On, D., Tazaki, M., Noji, A., Van Heck, G., De Vries, J., Sucre, J.A., Picard-Ami, L., Kabanov, M., Lomachenkov, A., Burkovsky, G., Carrasco, R.L., Bodharamik, Y., Meesapya, K., Skevington, S., Patrick, D., Martin, M., Wild, D., Acuda, W., Mutambirwa, J., Bonicaato, S., Yongping, G., Fleck, M., Angermeyer, M.C., Kilian, R., Kwok-fai, L., Billington, R., Bullinger, M., Kuyken, W., Sartorius, N. (1998). Development of the World Health Organization WHOQOL-BREF quality of life assessment. The WHOQOL Group. *Psychol Med* 28, 551–558.

Harris, P.A., Taylor, R., Minor, B.L., Elliott, V., Fernandez, M., O’Neal, L., McLeod, L., Delacqua, G., Delacqua, F., Kirby, J., Duda, S.N. (2019). The REDCap consortium: Building an international community of software platform partners. *J Biomed Inform* 95, 103208.

Hurst, N.P., Kind, P., Ruta, D., Hunter, M., Stubbings, A. (1997). Measuring health-related quality of life in rheumatoid arthritis: validity, responsiveness and reliability of EuroQol (EQ-5D). *Rheumatology* 36, 551–559.

Johnson, C. (2005). Measuring Pain. Visual Analog Scale Versus Numeric Pain Scale: What is the Difference? *J Chiropr Med* 4, 43–44.

Kirisci, L., Moss, H.B., Tarter, R.E. (1996). Psychometric evaluation of the Situational Confidence Questionnaire in adolescents: fitting a graded item response model. *Addictive Behaviors* 21, 303–317.

Korff, M. V., Deyo, R.A., Cherkin, D., Barlow, W. (1993). Back pain in primary care: Outcomes at 1 year. *Spine (Phila Pa 1976)* 18, 855–862.

Liu, H., Cella, D., Gershon, R., Shen, J., Morales, L.S., Riley, W., Hays, R.D. (2010). Representativeness of the PROMIS Internet Panel. *J Clin Epidemiol* 63, 1169.

Melzack, R. (1987). The short-form McGill Pain Questionnaire. *Pain* 30, 191–197.

Mollayeva, T., Thurairajah, P., Burton, K., Mollayeva, S., Shapiro, C.M., Colantonio, A. (2016). The Pittsburgh sleep quality index as a screening tool for sleep dysfunction in clinical and non-clinical samples: A systematic review and meta-analysis. *Sleep Med Rev* 25, 52–73.

Van Nispen, R.M.A., Knol, D.L., Langelaan, M., Van Rens, G.H.M.B. (2011). Re-evaluating a vision-related quality of life questionnaire with item response theory (IRT) and differential item functioning (DIF) analyses. *BMC Med Res Methodol* 11, 1–10.

Price, D.D., McGrath, P.A., Rafii, A., Buckingham, B. (1983). The validation of visual analogue scales as ratio scale measures for chronic and experimental pain. *Pain* 17, 45–56.

Rabin, R., De Charro, F. (2001). EQ-5D: a measure of health status from the EuroQol Group. *Ann Med* 33, 337–343.

Samejima, F. (1968). ESTIMATION OF LATENT ABILITY USING A RESPONSE PATTERN OF GRADED SCORES1. *ETS Research Bulletin Series* 1968, i–169.

Santos, J.G., Brito, J.O., de Andrade, D.C., Kaziyama, V.M., Ferreira, K.A., Souza, I., Teixeira, M.J., Bouhassira, D., Baptista, A.F. (2010). Translation to Portuguese and validation of the Douleur Neuropathique 4 questionnaire. *The Journal of Pain: Official Journal of the American Pain Society* 11, 484–490.

Santos, M., Cintra, M.A.C.T., Monteiro, A.L., Santos, B., Gusmão-Filho, F., Andrade, M.V., Noronha, K., Cruz, L.N., Camey, S., Tura, B., Kind, P. (2016). Brazilian Valuation of EQ-5D-3L Health States: Results from a Saturation Study. *Med Decis Making* 36, 253–263.

Timmerman, H., Steegers, M.A.H., Huygen, F.J.P.M., Goeman, J.J., Van Dasselaar, N.T., Schenkels, M.J., Wilder-Smith, O.H.G., Wolff, A.P., Vissers, K.C.P. (2017). Investigating the validity of the DN4 in a consecutive population of patients with chronic pain. *PLoS One* 12.

Underwood, M.R., Barnett, A.G., Vickers, M.R. (1999). Evaluation of two time-specific back pain outcome measures. *Spine (Phila Pa 1976)* 24, 1104–1112.

Ware, J.E., Kosinski, M., Keller, S.D. (1996). A 12-Item Short-Form Health Survey: construction of scales and preliminary tests of reliability and validity. *Med Care* 34, 220–233.
